# Examination of Physiological and Morphological Differences between Farm-Bred and Wild Black-Spotted Pond Frogs (*Pelophylax nigromaculatus*)

**DOI:** 10.3390/life11101089

**Published:** 2021-10-15

**Authors:** Jun-Kyu Park, Jeong Bae Kim, Yuno Do

**Affiliations:** 1 Department of Biological Science, Kongju National University, Gongju 32588, Korea; pjk8578@smail.kongju.ac.kr; 2 Inland Fisheries Research Institute, National Institute of Fisheries Science, Seoul 12453, Korea; jbkim347@korea.kr

**Keywords:** animal health, ex situ conservation, farm-bred and wild frog, nutritional status, veterinary clinical examination

## Abstract

**Simple Summary:**

We compared the health status and ecological characteristics of farm-bred and wild frogs using veterinary clinical examinations. Wild frogs showed a better nutritional status and superior traits of locomotory performance than farm-bred frogs. However, wild animals are not necessarily in good health. This comparison can help determine the condition of animals and suggest improvements to the breeding environment.

**Abstract:**

Due to the decline in the population and the difficulty of in situ conservation, several anuran species are being reared in captivity. In this study, we identified physiological and morphological differences between farm-bred and wild frogs. Nine different serum components were used as indicators of osmotic pressure, homeostatic state, organ function, and nutritional status of farm-bred frogs and wild frogs, while radiographic techniques were used to visualize differences in bone mineral density and body composition ratio. Additionally, X-ray skeletal images were used for morphological analysis to estimate differences in locomotory performance between the two groups. Wild frogs harbor traits that aid in better locomotory performance than farm-bred frogs. They also have a relatively lower fat content ratio and higher calcium and phosphorus serum levels than farm-bred frogs, suggesting a difference in nutritional status. However, hepatic stress was higher in wild frogs than in farm-bred frogs. Veterinary clinical examinations allow for the identification of differences in nutritional and morphological conditions between farm-bred and wild frogs. Determining the health of animals can help improve their living conditions, eliminate conditions that can negatively affect them, and effectively manage them on farms, in zoos, and at ex situ conservation institutes.

## 1. Introduction

The welfare and ethical treatment of animals are particularly important in the case of captive animals that are artificially bred for various purposes [1]. One important factor in animal welfare is ensuring that captive animals are healthy [2], and this requires suitable living conditions and quality care [3,4]. In fact, the health status of captive animals reared in a negative environment tends to be much worse than that of animals living in their natural habitat [3]. Rearing animals in a confined space or in a high density of individuals may result in the rapid spread of diseases [5]. Additionally, captive animals fed a less nutritious diet than wild animals may be subject to metabolic diseases or nutritional deficiencies [6]. Consequently, it is clear that poor rearing conditions can have a negative impact on the health and longevity of captive animals [7]. Therefore, it is important to be aware of the variations in physiological stress responses among different species depending on the ecological characteristics of the species or the type of rearing environment [8,9]. Moreover, to improve the living conditions and welfare of captive animals, it is necessary to evaluate the health of various captive animals according to the physiological characteristics of each species and subsequently identify factors that negatively affect the health status of the animals in question.

Several amphibian species worldwide are experiencing declining numbers and even extinction, and are consequently being bred in captivity. Therefore, identifying environmental factors that affect the health of frogs is particularly important. Some species that are sold as food or pets are raised according to local regulations or international conventions [10,11,12,13]. Other amphibian species that are difficult to preserve in their natural habitat (for example, because of the effects of the pandemic pathogen *Batrachochytrium dendrobatidis*) are preferentially selected and bred for ex situ conservation [14,15,16]. Amphibians have low maintenance requirements and are easy to breed in the laboratory because of their small body size, high fecundity, and short generation time. This makes them ideal candidates for ex situ conservation [17]. While many species of amphibians are bred for various purposes in good living conditions [18], these animals are often exposed to diseases and problems associated with captivity [15]. Additionally, the exploitation of amphibians for black market trade or commercial sale severely affects the amphibian population [19,20]. Thus, a standardized procedure for determining their health status and adequacy of their captive environment is essential.

Determining the health status of the animals and the adequacy of their living conditions can be achieved by understanding the physical, chemical, and biological differences of habitat status between captive and wild amphibians. Captive amphibians may have a smaller range of motion, less access to food, and be exposed to a different environment than wild amphibians [21,22]. Long-term exposure to these factors can negatively impact their nutritional and physiological status and cause diseases [6,15,23]. The health status of captive vertebrate animals can be determined through various established veterinary clinical examinations [24]. These can be safely performed without killing the animal and aid in estimating several physical, chemical, and biological parameters associated with their health [24,25,26,27]. Among these examinations, blood chemistry analysis can be used to identify nutritional status, osmotic pressure in the body, homeostasis, physiological stress, and organ function status [25,26]. These parameters function as indicators of the suitability of the food and nutrients supplied to the captive frogs, as well as of the physical and chemical environmental stressors to which they are exposed [6,28]. Another method by which nutritional or disease status can be determined is radiographic examination [21,29,30]. Recently, it has been proposed that food intake or nutritional status can be determined from objective numerical values obtained from body composition or measurements of frogs [31,32]. Additionally, X-ray skeletal images of frogs can be used in identifying differences in ecological effects related to skeleton morphology, such as predation, microhabitat, or locomotory performance [33,34,35,36].

In this study, we compared physiological and morphological parameters between farm-bred frogs and wild frogs to determine the impact of living conditions on the health status of the frogs in question. Serum chemistry and radiographic analyses were used to estimate the nutritional and physiological status of the animals. Additionally, skeletal images were analyzed to assess the range of locomotion and predatory pressure experienced by the frogs. These criteria were used as indicators of the quality of living environment and were compared between the wild frogs and the frogs bred in captivity. A comparison between males and females was also conducted to confirm whether the sex of the animal is relevant when determining its health. The examination protocol developed in this study can be adopted to monitor and improve the living conditions of frogs reared in captivity.

## 2. Materials and Methods

### 2.1. Experimental Animal

From May 2019 to June 2020, 50 black-spotted pond frogs (*Pelophylax nigromaculatus*) were purchased from two different farms (farm-bred frogs). Farm-bred frogs were autonomously fed only crickets and mealworms, and they bred or hibernated in a greenhouse space (area of 1500–2000 m^2^) narrower than their wild habitats. Both farms started farming captive frogs in 2003 and 2005, respectively. Additionally, both hibernation and breeding take place within the greenhouse. Fifty black-spotted pond frogs (*Pelophylax nigromaculatus*) were captured from Chungcheongnam-do between 9 pm and 11 pm in May 2019 and June 2019, and again in June 2020 and July 2020 (wild frogs). Wild frogs were collected from four different rice paddy fields around reservoirs, rivers, and marshes to eliminate differences caused by local environmental variations. There were no factors that could stress the frogs, such as chemicals, noise, or light pollution around the collection sites. The appearance of the frogs was scrutinized to confirm that there were no abnormalities such as wounds, ascites, swellings, or deformities. Both farm-bred and wild frogs were confirmed to have no abnormalities in appearance after an acclimation period of 2 h. Both groups of frogs contained breeding and non-breeding individuals; however, only mature frogs with secondary sexual characteristics were used for analysis. Juvenile frogs exhibit high growth rates and could potentially contribute to large variations in the analysis; thus, they were excluded from the analysis. Males were distinguished by the presence of the nuptial pad on the front toe and the vocal sac. Adults without both characteristics were considered female. Therefore, frogs that were larger than the size of the collected males and had no male characteristics were judged to be female. Other individuals were judged as immature juvenile frogs and excluded from the analysis. In total, 35 farm-bred frogs (18 males and 17 females) and 36 wild frogs (18 males and 18 females) were assessed in the physiological and morphological analyses. Of the frogs not used in the analysis, 14 wild frogs were released to their original sampling sites, and 15 farm-bred frogs were bred in the Zoology Lab of Kongju National University. In total, 71 frogs (35 farm-bred and 36 wild frogs) were euthanized after blood sample collection for further analysis by spinal cord pithing, and fixed in 70% ethanol. Experimental procedures were performed in accordance with the regulations of and with the approval of the Experimental Animal Ethics Committee of Kongju National University (KNU_2019-01).

We measured the snout-vent length (SVL) in the order of 0.01 mm units using a digital caliper. This measurement is an indicator of amphibian body size. Body weights were also measured using a digital balance in the order of 0.01 g units. These measurements were used to confirm the difference in basic physical condition between farm-bred and wild frogs as well as between female and male frogs.

### 2.2. Serum Extraction and Blood Chemistry Analysis

Chemical anesthesia can affect the muscle physiology in frogs [37]. Therefore, its use would impact our ability to analyze the muscles of the frogs. However, freezing with ice-cold water can be used as anesthesia for ectothermic vertebrates such as amphibians and reptiles [38]. Since we wanted to analyze the muscles after blood extraction, blood samples (equivalent to 1% of each frog’s weight) were collected from frogs anesthetized in ice-cold water by cardiac venipuncture within 2 h of acclimatization. Blood samples were harvested in a serum separator tube and centrifuged (3000× *g*, 10 min) to obtain serum. All samples were stored at −40 °C until further analysis.

A clinical chemistry automated analyzer (Hitachi Automatic Analyzer 7020, Hitachi High-Technologies, Tokyo, Japan) was used to analyze nine serum components, namely total protein (TP), albumin, glucose, calcium, phosphorus, alanine aminotransferase (ALT), aspartate aminotransferase (AST), blood urea nitrogen (BUN) [17], and creatinine. TP and albumin are conventionally used as indicators of nutritional, protein metabolism, homeostatic, renal, and hepatic status [23,28]. Glucose, calcium, and phosphorus are used as indicators of the nutritional status of frogs [23]. ALT and AST are used as indicators of hepatic stress [28], while BUN is an indicator of protein metabolism as it is a waste product of that process [39]. Creatinine is an indicator of muscle metabolism as it derives from creatine and glucose, which are used as energy sources for muscle metabolism [40,41]. Additionally, simultaneous increases in BUN and creatinine levels are indicators of kidney dysfunction [39].

### 2.3. Measurement of Body Composition and BMD

**Figure 1:**
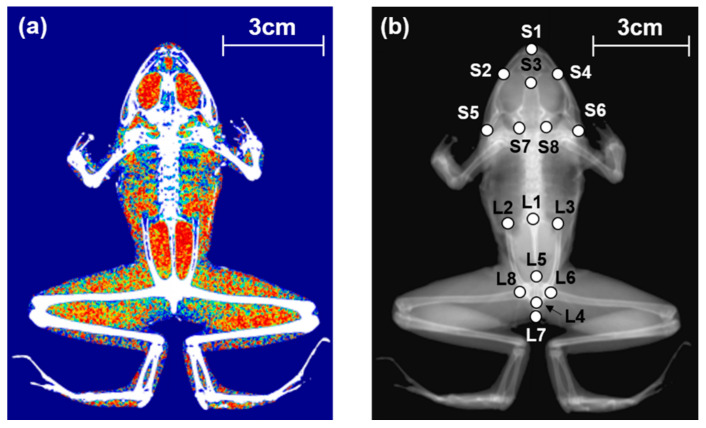
X-ray image of female wild frog (*Pelophylax nigromaculatus)* from dual energy X-ray absorptiometry: (**a**) body composition image (red areas: fat contents, green areas: lean body contents) (**b**) body image with 16 landmark points. Landmark points characterize the skull and lower body (ilium, pelvis, urostyle): (S1) the posterior tip of the premaxilla, (S2) the left tip of the maxilla, (S3) the anterior tip of the sphenethmoid, (S4) the right tip of the maxilla, (S5) the left tip of the quadrate, (S6) the right tip of the quadrate, (S7) the posterior left tip of the prootic, and (S8) the posterior right tip of the prootic (L1) the anterior tip of the urostyle, (L2) the anterior left tip of the ilium, (L3) the anterior right tip of the ilium, (L4) the posterior tip of the urostyle, (L5) anterior tip of the pelvis, (L6) the posterior right tip of the pelvis, (L7) the posterior tip of the ischium, and (L8) the posterior left tip of the pelvis.

Dual energy X-ray absorptiometry (DEXA; Medikors InAlyzer, Seongnam, Korea) was used to measure body composition and BMD. DEXA helps calculate bone mineral content (BMC), fat content, and lean body content with the use of alternately transmitting high-and low-energy X-rays (Figure 1a). Of these three components, lean body content is usually calculated as the sum of water and muscle content [42]. We processed tissue samples for at least 3 months in 99.5% methanol to exclude body water and considered the measured lean body mass as muscle mass. Three types of images (body image, bone image, and composition image) were acquired using DEXA. Body images were used to compare the skeletal shape and length of the bone between females and males, and farm-bred and wild frogs. Percent BMC, fat content, lean body content, and BMD were used to compare females and males, as well as farm-bred and wild frogs.

### 2.4. Analysis of Skeletal Shape

We used landmark-based geometric morphometrics to analyze skeletal shape. TpsDig software [43] was used to digitize the landmark points for the shape of the skull and lower body. A total of eight landmarks were designated in the skull of frogs. Another eight landmarks were designated in the lower body of the frogs (Figure 1b). Morpho J software (version 1.07a, Manchester, UK) was used to convert digitized landmark coordinates into procrustes coordinate values and to compare variations in the skeletal shape among farm-bred female, farm-bred male, wild female, and wild male frogs. Canonical variate analysis (CVA) was used to compare the morphological differences in the skeletal shape in skull and lower body among each group. A rectangular grid with the landmark vectors and a wireframe graph of canonical variates 1 (CV1) and 2 (CV2) axes were employed to visualize the difference in the skeletal shape obtained from each group. The significantly different skeletal shape among the four groups was confirmed by Mahalanobis distance of CVA and *p*-value of this distance. We also used a graph that matched the average skeletal shape of the four groups with an average skeletal shape of each group, and compared the morphological distance.

### 2.5. Statistical Analysis

A two-way ANOVA test was used to compare the difference of each content (SVL, body weight, serum components, body composition, and BMD) between farm-bred and wild frogs, and between male and female frogs. Two-way ANOVA was employed to identify interaction effects between the two main factors (Group*Sex). If interaction effects were present, Tukey’s post hoc test were performed on each group (farm-bred female, farm-bred male, wild female, and wild male). Statistical analyses were performed by GraphPad Prism version 7.0 for Windows (GraphPad Software, San Diego, CA, USA). Morphological differences in skeletal shape between females and males and between farm-bred and wild frogs were analyzed by CVA in Morpho J. All statistical differences were considered to be significant at *p* < 0.05.

## 3. Results

### 3.1. Basic Physical, Nutritional, and Physiological Status of Frogs

**Figure 2:**
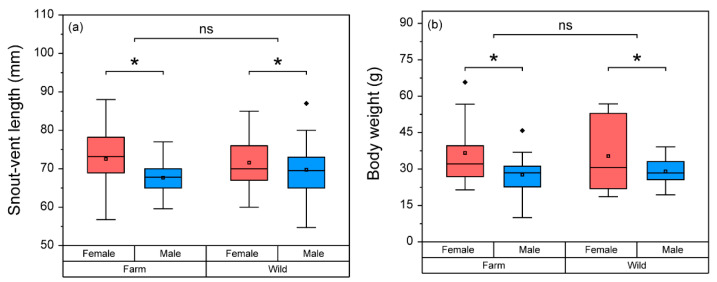
A comparison of the snout-vent length (**a**) and body weights (**b**) between the two main factors (group, farm–wild; and sex, female–male). Box plots show the mean (central square dot), median (central band), 25th and 75th percentiles (bottom and top of boxes), 1.5 interquartile range (bottom and top of line), outlier (diamonds dots). Because a significant interaction effect was not observed in a two-way ANOVA test, significant differences (*p* < 0.05) were determined in each main factor and are represented with asterisks (*).

Neither SVL (F = 0.819) nor body weight (F = 0.296) were significantly different (*p* > 0.05) between the two main groups (Group*Sex). The frogs did not significantly differ (*p* > 0.05) in SVL (F = 0.111) or body weight (F = 5.161 × 10^−6^) between the farm and wild groups. However, SVL (F = 4.161) and body weight (F = 9.279) of female frogs were significantly higher (*p* < 0.05) than in male frogs (Figure 2).

We determined the levels of nine serum components to compare the nutritional and physiological status of farm-bred and wild frogs, as well as the differences between female and male frogs (Table 1). The serum levels of ALT and TP showed a significant interaction effect with the two main factors, whereas no significant interaction effects were observed with the other seven serum components (Table 2).

Glucose, BUN, and creatinine did not significantly differ (*p >* 0.05) between groups (farm-wild) or between sexes.

AST of wild frogs was significantly higher (*p* < 0.05) than that of farm-bred frogs, but this was not the case between sexes (*p >* 0.05). The wild frogs had significantly higher levels (*p* < 0.05) of ALT than farm-bred frogs, and the female frogs had significantly higher levels (*p* < 0.05) of ALT than male frogs.

Notably, the ALT of wild female frogs had the highest significance (*p* < 0.05) among the four groups, whereas the ALTs of the other three groups did not significantly differ (*p* > 0.05).

TP and albumin values were not significantly different (*p* > 0.05) between farm-bred frogs and wild frogs, whereas the female frogs had significantly higher (*p* < 0.05) TP and albumin levels than the male frogs.

Wild frogs had significantly higher (*p* < 0.05) serum levels of calcium and phosphorus than the farm-bred frogs. The female frogs had significantly higher (*p* < 0.05) calcium levels than male frogs, whereas phosphorus did not significantly differ (*p* > 0.05) between the sexes.

### 3.2. Comparison of Body Composition and BMD among Frogs

**Figure 3:**
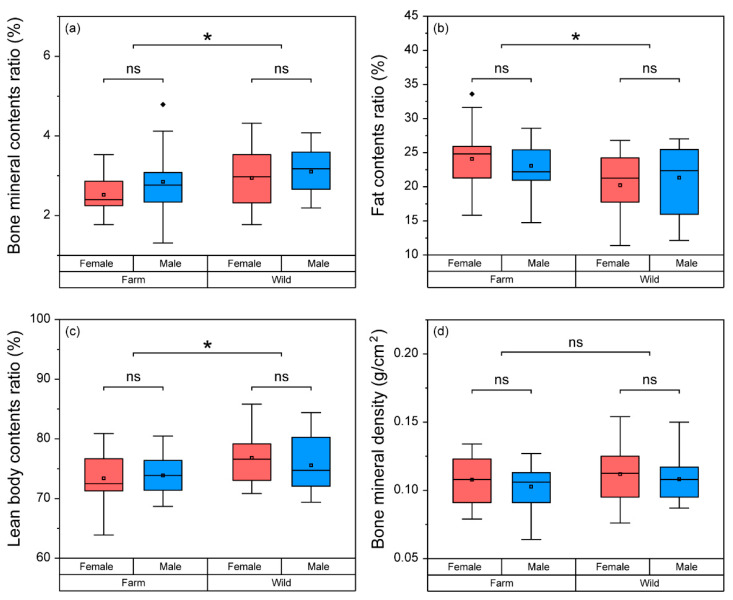
A comparison of body composition (bone mineral contents; (**a**) fat contents; (**b**) lean body contents; (**c**) and ratio and bone mineral density (**d**) between the two main factors (group; farm–wild, and sex; female–male). Box plots show mean (central square dot), median (central band), 25th and 75th percentiles (bottom and top of boxes), 1.5 interquartile range (bottom and top of line), outlier (diamonds dots). Because there was no significant interaction effect observed for the main factors in the two-way ANOVA test, significant differences (*p* < 0.05) were determined in each main factor and are represented with asterisks (*).

BMC (F = 0.293), fat contents (F = 1.063), lean body contents (F = 0.756) ratios, and BMD (F = 0.028) did not have a significant interaction effect (*p* > 0.05) in the two main factors (Group*Sex). Wild frogs had a significantly higher (*p* < 0.05) BMC (F = 4.485) and lean body contents (F = 6.675) ratios, and a lower fat contents ratio (F = 7.347) than farm-bred frogs, but there was no significant difference in BMD (F = 1.235, *p* > 0.05) between the two groups (Figure 3). Meanwhile, the differences in BMC (F = 2.318), fat contents (F = 0.001), lean body contents (F = 0.155) ratios, and BMD (F = 1.061) were not significant (*p* > 0.05) between female and male frogs.

### 3.3. Skeletal Morphology

**Figure 4:**
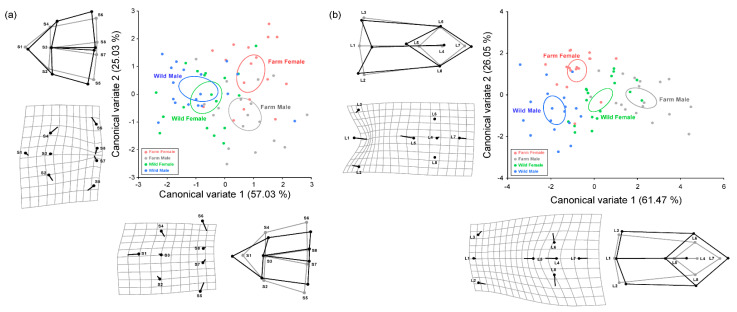
Scatterplot composed of canonical variates 1 (CV1) and 2 (CV2) axes, thin-plate spline deformation grids with landmarks, and wireframe graph to explain morphological difference in (**a**) skull and (**b**) skeleton of lower body (ilium, pelvis, urostyle) among farm-bred female frogs, farm-bred male frogs, wild female frogs, and wild male frogs from canonical variate analysis (CVA). Circles in the deformation grids and the gray wireframe graphs indicate the skeletal shape of the individuals with a lowest CV value in each CV axis. Pins in the deformation grids and the black wireframe graphs indicate the change in skeletal shape by an increase in CV value in each CV axis.

CVA was used to analyze morphological differences among the four groups (farm female frogs, farm male frogs, wild female frogs, and wild male frogs). In the skull shape analysis, CV1 accounted for 57.03% of morphological variance, and individuals with a higher CV1 represented a longer and narrower skull shape. CV2 accounted for 25.03% of morphological variance, and the individuals with a higher CV2 represented a shorter and wider skull shape (Figure 4a). Farm-bred frogs of both sexes had a higher value of CV1 than wild frogs, demonstrating that frogs bred in captivity had a narrower and longer skull shape than wild frogs.

In the skeletal analysis of the lower body, CV1 accounted for 61.47% of morphological variance, where the individuals with a higher CV1 represented a wider pelvis with shorter ilium and urostyle. CV2 accounted for 26.05% of morphological variance, and the individuals with a higher CV2 represented a longer and narrower pelvis with shorter urostyle (Figure 4b). The frogs from the four groups were divided by CV1 axis. Wild males had the lowest CV1 values of all the groups, and farm-bred males had the highest. The farm-bred female frogs were separated from the other groups on the CV2 axis.

**Figure 5:**
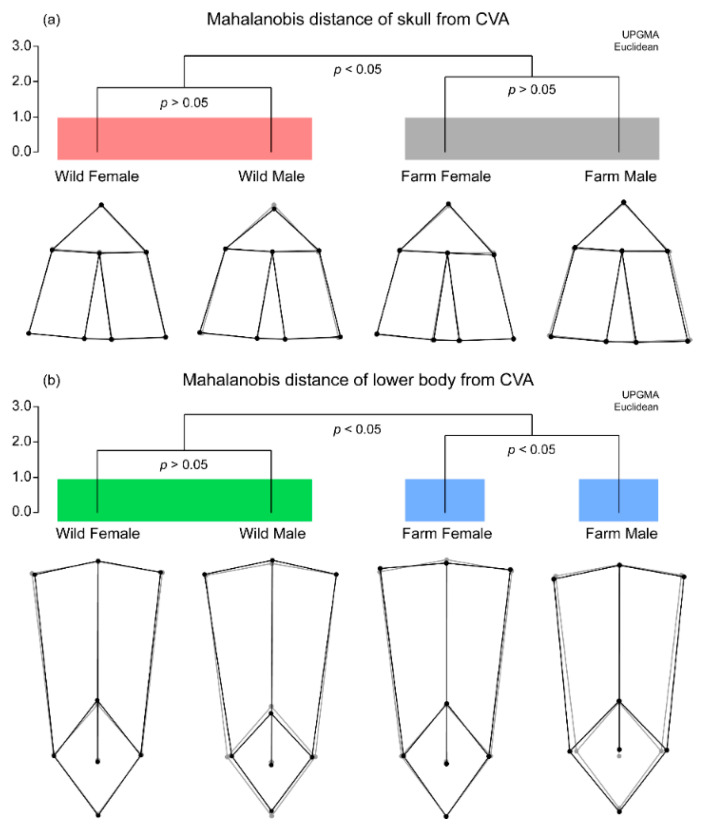
Mahalanobis distance of skeletal shape among four groups obtained by CVA. The distance is represented by an unweighted pair group method with an arithmetic mean (UPGMA) hierarchical tree (Euclidean). The gray wireframe graphs represent mean skeletal shape of four groups, and the black wireframe graphs represent mean skeletal shape of each group. (**a**) mahalanobis distance and *p*-value for skull shape (**b**) mahalanobis distance and *p*-value for skeletal shape of lower body.

We confirmed the significance of morphological differences among four groups by calculating Mahalanobis distance in CVA (Figure 5). In the morphological analysis of skull shape, a significant morphological distance was found between farm-bred and wild frogs. However, there was no significant difference between female and male frogs within each group. The wild female frogs had a skull shape similar to the average shape of the four groups, while the wild male frogs had a short and wide skull shape. In contrast, the farm-bred female frogs had a longer skull shape than the average shape of the groups, and the farm-bred male frogs had a narrower skull shape.

Likewise, there were differences between farm-bred and wild frogs in the morphological analysis of skeletal shape of the lower body. However, there was no significant difference between female and male wild frogs. Contrarily, the farm-bred female frogs and farm-bred male frogs showed a significant difference in lower body shape. Wild female frogs had a longer pelvis with a longer urostyle. Similarly, the wild male frogs had a narrower pelvis with longer urostyle. Farm-bred female frogs had a similar lower body shape to the average shape of the four groups, and farm-bred male frogs had a wider pelvis with shorter urostyle.

## 4. Discussion

In this study, nutritional, physiological, and morphological differences were compared between farm-bred and wild frogs. Some serum components differed between male and female frogs. AST and ALT, indicators of hepatic function, were higher in wild frogs than in farm-bred frogs. Calcium and the phosphorus levels in wild frogs were higher than those in farm-bred frogs, suggesting differences in the composition of diet and nutrients consumed by both groups. Body composition did not differ between males and females, while the farmed-bred frogs had a higher fat contents ratio and a lower lean body contents ratio than wild frogs. Although skull shape was not different between males and females, there was a significant difference between wild frogs and farm-bred frogs. Similarly, the skeletal shape of the lower body of wild frogs was significantly different from farm-bred frogs. In the farm-bred frogs, lower body shape was also significantly different between farm-bred female frogs and male frogs. These differences indicate that the body shape of wild frogs suggests a better jumping performance than the body shape of farm-bred frogs.

Wild frogs generally require better locomotory performance than farm-bred frogs [22,44]. This is a result of the difference between confined captive spaces and the distance between breeding and the hibernating sites for wild frogs. Moreover, when the required locomotory performance differs between groups, their environmental and energy requirements may also be dissimilar [45,46]. In addition, the ability to escape from predators is determined by locomotion performance, which in turn is dependent on skeletal shape. Consequently, better locomotion performance has a positive effect on predator avoidance [46,47]. Over generations, animals raised in captivity develop a lower level of evasion response to predators [44]. The black-spotted pond frogs used in our study are jumper frogs that do not use camouflage or poison, and the narrower pelvis and long tailbone of the wild frogs can act as traits for improved jumping performance [33,34]. These differences in locomotory performance may be caused by the higher predatory pressure experienced in wild populations and their ability to find and move to new breeding sites. Additionally, the skeletal or external shape is affected at a specific stage of the frog life cycle by biotic and abiotic factors [34,48]. This phenotypic change also occurs in the skull. Changes in the timing of metamorphosis caused by the presence of predators can cause the shape of the head and body to widen or narrow [48]. Unlike farm-bred frogs, wild populations may be exposed to different environments throughout their entire life cycle, and this seems to result in a different skeletal shape. In particular, the difference between male and female within the group that did not appear in the skull shape, appeared in the skeletal shape of lower body between male and female of the farm-bred frogs. Farm-bred male frogs had traits suggestive of the lowest locomotor performance. Male frogs are more mobile during the breeding season to migrate to breeding grounds, defend their territory, and mate with females [49,50]. This behavior may be relatively less necessary in a farm environment with confined space. In our results, the different responses of farm-bred and wild male frogs are likely due to the combination of these ecological traits and environmental conditions. To sum up, the necessity of exploration for breeding sites and avoiding predators is lower in farm-bred frogs, and the biotic and abiotic environment can be relatively stable during their life history. Therefore, this population does not develop a skeletal shape associated with higher locomotory performance.

The fat content of farm-bred frogs was higher than that of wild frogs. This may be due to differences in locomotory performance or food consumed between farm-bred and wild frogs. Differences in the locomotory performance of frogs can change their energy requirements [45], and consequently change the levels of stored fat [51]. A poorer locomotory performance is sufficient for farm-bred frogs to live in a narrower space range, and it results in the accumulation of fat in the body. Additionally, fat content can be changed by overfeeding or nutrient imbalance [6,23]. In general, captive individuals tend to have a narrower diet spectrum than wild individuals [3,22], and this is true for our study as well. The combination of these environmental conditions may have contributed to the differences in body composition between farm-bred and wild frogs. Likewise, the calcium and phosphorus levels in blood were higher in wild frogs, which also appeared to be due to differences in the composition of diet and nutrients accessible to the two groups. Calcium and phosphorus in food are essential for calcium metabolism and skeletal development, and a deficiency or abundance can lead to various diseases, such as metabolic bone disease and secondary nutritional hyperparathyroidism [6,23]. Improving living conditions, such as by expanding the spatial range, dusting nutrients in food, and/or supplying various foods, can help to prevent nutritional deficiencies or imbalances.

Contrary to nutritional status, AST and ALT levels, which are indicators of the hepatic state, were higher in wild frogs. Wild animals are exposed to several stressors, such as physical, chemical, and biological stresses. Examples include traffic noise, exposure to salinity, and predation. Exposure to these triggers the release of corticosterone, a stress hormone in amphibians [52,53,54]. High levels of corticosterone resulting from chronic stress can lead to changes in hepatic condition and function through increasing the glucose metabolism of liver [55,56,57,58]. In fact, animals that had been reared in captivity for long periods were found to exhibit lower levels of stress indicators, such as rates of infection with pathogens and parasites, compared to recently captured animals [15]. Infection with these parasites or pathogens also acts as a stressor that increases corticosterone [59]. Additionally, wild frogs are exposed to many environmental stressors that can directly affect liver metabolism, such as toxic chemicals used in agriculture [60,61]. These biotic and abiotic stresses are more likely to impact wild animals living in various environments than farm-bred animals that are protected and cared for in stable indoor aquariums [8]. This shows that wild frogs may not be healthier than farm-bred frogs in all aspects, such as liver function.

Various types of veterinary examination can help improve the inadequate captive environment by making it possible to predict the exact condition of the individual. However, some serum components (ALT, TP, albumin, and calcium) showed differences between males and females. This corroborates previous studies [62,63] and suggests that it is necessary to consider the sex of the animal when veterinary clinical examinations are used to determine the health of amphibians. Interestingly, our results showed that sexual-specific responses may also differ between farm-bred and wild frogs. The ALT of wild female frogs was far higher than that of the other three groups. Although ALT is a very sensitive indicator compared to AST [28], high variation values of glucose and AST suggest that hepatic stress is present in wild female frogs. Female frogs that produce and store eggs must expend more energy on reproduction than male frogs [64,65]. Moreover, they show more extreme and distinct changes in the energy metabolism of lipids, proteins, and glycogen in the liver compared to male frogs [66,67,68]. We think that this combination of sex-specific energy metabolism in the liver and various environmental stresses in the wild may exacerbate hepatic stress in wild female frogs. To diagnose various stressors or health conditions of individuals using this analysis, physiological reference intervals (RIs) according to various environmental conditions such as season, sex, and life history are required. Although we have previously established RIs of body composition and serum components for male black-spotted pond frogs [69], these RIs were not used in the present study because there was a comparison group and there were no RIs for females. However, the establishment of RIs is sufficiently meaningful in that a comparison group is not necessary, and because it can be used for diagnosis on an individual unit [26,70]. To diagnose a captive individual without a comparison group, we suggest that the establishment of RIs in veterinary clinical examinations in captive and wild frogs is necessary. On the other hand, we analyzed the physiological and morphological changes without identifying the generations of farm-bred and wild frogs. The factors we measure are those that can change more within a specific period of one generation than across several generations. [48,69]. Later, studies of factors that occur across generations, such as changes in the gene pool, may be necessary. The accumulation of such compositive information can help us to investigate the condition of captive and breeding animals in more detail.

## 5. Conclusions

We confirmed how the physiological and morphological adaptive responses in farm-bred frogs differ from those in wild frogs. Morphological analysis and physiological analysis helped us to predict how the nutritional and spatial conditions of farm frogs and wild frogs differ. Although some health-related components seemed to be better in wild frogs, the health conditions of frogs from wild populations were not unconditionally better than those of farm-bred frogs. Understanding the health condition of frogs and improving the environment that may negatively affect them can aid in promoting more efficient animal management and environmental enrichment. These examinations will be suitable for use on farms and in zoos, as well as in ex situ conservation institutions to diagnose the health status of individuals and to secure healthy animals.

## Figures and Tables

**Table 1:** Mean ± standard deviation (SD) of nine serum components (glucose, aspartate aminotransferase; AST, alanine aminotransferase; ALT, creatinine, blood urea nitrogen; BUN, total protein; TP, albumin, calcium, and phosphorus) from four groups (farm female frogs, farm male frogs, wild female frogs, and wild male frogs).

Contents	Farm		Wild	
	Female	Male	Female	Male
Glucose (mg/dL)	21.75 ± 8.98	19.13 ± 9.60	21.74 ± 24.69	24.71 ± 17.70
AST (U/L)	283.27 ± 230.15	305.23 ± 254.23	533.90 ± 346.96	389.44 ± 268.94
ALT (U/L)	51.25 ± 66.58	56.14 ± 58.82	120.73 ± 81.79	59.39 ± 54.42
BUN (mg/dL)	13.83 ± 17.01	17.00 ± 18.47	12.04 ± 10.69	10.32 ± 11.62
Creatinine (mg/dL)	0.15 ± 0.08	0.14 ± 0.07	0.14 ± 0.05	0.10 ± 0.05
TP (g/dL)	3.32 ± 0.84	2.50 ± 0.63	3.80 ± 0.80	2.04 ± 0.37
Albumin (g/dL)	0.94 ± 0.26	0.69 ± 0.21	1.00 ± 0.26	0.56 ± 0.11
Calcium (mg/dL)	10.05 ± 4.39	6.08 ± 1.37	14.17 ± 6.66	6.56 ± 1.46
Phosphorus (mg/dL)	6.76 ± 2.87	6.55 ± 3.10	11.05 ± 4.58	9.32 ± 3.29

**Table 2:** Comparison of the serum components using the two-way ANOVA test between two main factors (group, farm–wild; and sex, female–male). In the contents with significant interaction effects of main factors from two-way ANOVA test, Tukey’s post hoc test was performed among four groups (farm female frogs, FF; farm male frogs, FM; wild female frogs, WF; and wild male frogs, WM).

Contents	Main Factor	df	Mean Square	F Value	*p*-Value	Summary
Glucose (mg/dL)	Group	1	137.594	0.497	0.483	Farm = Wild
	Sex	1	0.526	0.002	0.965	Female = Male
	Group*Sex	1	138.078	0.499	0.482	NS
AST (U/L)	Group	1	497,215.951	6.379	0.014	Farm < Wild
	Sex	1	66,557.517	0.854	0.359	Female = Male
	Group*Sex	1	122,821.288	1.576	0.214	NS
ALT (U/L)	Group	1	23,455.741	5.348	0.024	Farm < Wild
	Sex	1	14,130.106	3.222	0.077	Female > Male
	Group*Sex	1	19,457.491	4.437	0.039	WF > FF, FM, WM
BUN (mg/dL)	Group	1	317.233	1.450	0.233	Farm = Wild
	Sex	1	9.311	0.043	0.837	Female = Male
	Group*Sex	1	105.807	0.484	0.489	NS
Creatinine (mg/dL)	Group	1	0.012	2.680	0.106	Farm = Wild
	Sex	1	0.011	2.452	0.122	Female = Male
	Group*Sex	1	0.004	0.920	0.341	NS
TP (g/dL)	Group	1	0.002	0.004	0.951	Farm = Wild
	Sex	1	29.546	63.117	<0.001	Female > Male
	Group*Sex	1	3.854	8.233	0.005	FF, WF > FM, WM
Albumin (g/dL)	Group	1	0.023	0.490	0.486	Farm = Wild
	Sex	1	2.103	44.859	<0.001	Female > Male
	Group*Sex	1	0.166	3.544	0.064	NS
Calcium (mg/dL)	Group	1	93.803	5.554	0.021	Farm < Wild
	Sex	1	594.782	35.218	<0.001	Female > Male
	Group*Sex	1	59.233	3.507	0.065	NS
Phosphorus (mg/dL)	Group	1	221.103	17.714	<0.001	Farm < Wild
	Sex	1	16.790	1.345	0.250	Female = Male
	Group*Sex	1	10.199	0.817	0.369	NS
